# Why public health matters today and tomorrow: the role of applied public health research

**DOI:** 10.17269/s41997-019-00196-2

**Published:** 2019-03-18

**Authors:** Lindsay McLaren, Paula Braitstein, David Buckeridge, Damien Contandriopoulos, Maria I. Creatore, Guy Faulkner, David Hammond, Steven J. Hoffman, Yan Kestens, Scott Leatherdale, Jonathan McGavock, Wendy V. Norman, Candace Nykiforuk, Valéry Ridde, Janet Smylie

**Affiliations:** 1 University of Calgary, Calgary, Canada; 2 University of Toronto, Toronto, Canada; 3 McGill University, Montreal, Canada; 4 University of Victoria, Victoria, Canada; 5 CIHR Institute of Population & Public Health and University of Toronto, Toronto, Canada; 6 University of British Columbia, Vancouver, Canada; 7 University of Waterloo, Waterloo, Canada; 8 CIHR Institute of Population & Public Health and York University, Toronto, Canada; 9 Université de Montréal, Montreal, Canada; 10 University of Manitoba and the Children’s Hospital Research Institute of Manitoba, Winnipeg, Canada; 11 University of British Columbia, Vancouver, Canada; 12 University of Alberta, Edmonton, Canada; 13 IRD (French Institute For Research on Sustainable Development), CEPED (IRD-Université Paris Descartes), ERL INSERM SAGESUD, Université Paris Sorbonne Cités, Paris, France; 14 University of Montreal Public Health Research Institute (IRSPUM), Montreal, Canada

**Keywords:** Public health, Population health, Applied research, Policy, Sustainability, Equity, Santé publique, Santé des populations, Recherche appliquée, Politique (principe), Viabilité, Équité

## Abstract

Public health is critical to a healthy, fair, and sustainable society. Realizing this vision requires imagining a public health community that can maintain its foundational core while adapting and responding to contemporary imperatives such as entrenched inequities and ecological degradation. In this commentary, we reflect on what tomorrow’s public health might look like, from the point of view of our collective experiences as researchers in Canada who are part of an Applied Public Health Chairs program designed to support “innovative population health research that improves health equity for citizens in Canada and around the world.” We view applied public health research as sitting at the intersection of core principles for population and public health: namely sustainability, equity, and effectiveness. We further identify three attributes of a robust applied public health research community that we argue are necessary to permit contribution to those principles: researcher autonomy, sustained intersectoral research capacity, and a critical perspective on the research-practice-policy interface. Our intention is to catalyze further discussion and debate about why and how public health matters today and tomorrow, and the role of applied public health research therein.

## Résumé

La santé publique est essentielle à une société saine, juste et durable. Pour donner forme à cette vision, il faut imaginer une communauté de la santé publique capable de préserver ses valeurs fondamentales tout en s’adaptant et en réagissant aux impératifs du moment, comme les inégalités persistantes et la dégradation de l’environnement. Dans notre commentaire, nous esquissons un portrait possible de la santé publique de demain en partant de notre expérience collective de chercheurs d’un programme canadien de chaires en santé publique appliquée qui visent à appuyer « la recherche innovatrice sur la santé de la population en vue d’améliorer l’équité en santé au Canada et ailleurs ». Nous considérons la recherche appliquée en santé publique comme se trouvant à la croisée des principes fondamentaux de la santé publique et des populations, à savoir : la durabilité, l’équité et l’efficacité. Nous définissons aussi les trois attributs d’une solide communauté de recherche appliquée en santé publique nécessaires selon nous au respect de ces principes : l’autonomie des chercheurs, une capacité de recherche intersectorielle soutenue et une perspective critique de l’interface entre la recherche, la pratique et les politiques. Nous voulons susciter des discussions et des débats approfondis sur l’importance de la santé publique pour aujourd’hui et pour demain et sur le rôle de la recherche appliquée en santé publique.

## Introduction

Public health is critical to a healthy, fair, and sustainable society. Public health’s role in this vision stems from its foundational values of social justice and collectivity (Rutty and Sullivan 2010) and—we argue—from its position at the interface of research, practice, and policy.

Realizing this vision requires imagining a public health community that can maintain that foundational core, embrace opportunities of our changing world, and predict and adapt to emerging challenges in a timely manner. Unprecedented ecosystem disruption creates far-reaching health implications for which the public health community is unprepared (CPHA 2015; Whitmee et al. 2015). Human displacement is at its highest levels on record; those forced from home include “stateless people,” who are denied access to basic rights such as education, health care, employment, and freedom of movement (http://www.unhcr.org/figures-at-a-glance.html). Significant growth in urban populations creates an urgent need to improve urban environments, including policies to reduce air pollution and prevent sprawl (CPHA 2015; Frumkin et al. 2004), to reduce the substantial burden of morbidity and mortality attributable to behaviours such as physical inactivity, which negatively impact quality and quantity of life (Manuel et al. 2016). Significant and entrenched forms of economic, social, political, and historical marginalization and exclusion (TRC 2015), coupled with inequitable and unsustainable patterns of resource consumption and technological development (CPHA 2015; Whitmee et al. 2015), cause and perpetuate health inequities. These inequities underlie the now longstanding recognition that the unequal distributions of health-damaging experiences are the main determinants of health (CSDH 2008; Ridde 2004).

These imperatives demand a broadly characterized public health community. A now classic definition of public health is *the science and art of preventing disease, prolonging life, and promoting health through the organized efforts of society* (Last 2001). Public health, conceptualized in this manner, engages multiple sectors, embraces inclusion and empowerment (Ridde 2007), and demands navigating diverse political and economic agendas. Across Canada, a large and growing proportion of provincial spending is devoted to health care, while the proportion devoted to social spending (i.e., the social determinants of health) is small, flat-lining, and in some places declining (Dutton et al. 2018). Recent discourse has highlighted a weakening of formal public health infrastructure (Guyon et al. 2017) and points of fracture within the field (Lucyk and McLaren 2017). Efforts to strengthen public health, in its broadest sense, and to work towards unity of purpose (Talbot 2018) are needed now more than ever. What might such efforts look like?

We reflect on this question from our perspectives as researchers who are part of an Applied Public Health Chairs (APHC) program designed to support “innovative population health research that improves health equity for citizens in Canada and around the world.”^1^ The *applied* dimension^2^ is facilitated through the program’s focus on “interdisciplinary collaborations and mentorship of researchers and decision makers in health and other sectors” (http://www.cihr-irsc.gc.ca/e/48898.html). The APHC program (Box 1) is part of a broader set of efforts to address gaps in public health capacity, including research. Cross-cutting themes for the 2014 cohort (Box 2) include the following: healthy public policy, supportive environments (e.g., cities), diverse methodological approaches, global health, and health equity; many of which^3^ align with a Public Health Services and Systems Research perspective in that they “identif[y] the implementation strategies that work, building evidence to support decision-making across the public health sphere” (http://www.publichealthsystems.org/). Applied public health research is broad and could span CIHR Pillars 4 (social, cultural, environmental, and population health research) and 3 (health services research); the 2014 APHC cohort is predominantly aligned with Pillar 4.

The APHC program represents a significant Canadian investment in public health, and thus provides an important vantage point from which to reflect on why public health matters today, and tomorrow.

**Box 1** The Applied Public Health Chairs program

**Table:** 

Program objectives	
• Support high-quality programs of population health intervention research	
• Stimulate the application of innovative theories, methods and approaches in research and knowledge translation that promote reciprocal learning within and between countries	
• Catalyze interdisciplinary and intersectoral collaborations between researchers and knowledge users that contribute to evidence-informed decision-making and use of knowledge by public health and other sectors	
• Mentor the current and next generation of population and public health researchers, practitioners, and policy-makers from a range of disciplines and sectors	
Funding partners	
• CIHR Institute of Population & Public Health (*lead*)	
• Public Health Agency of Canada (*lead*)	
• CIHR Institute of Health Services & Policy Research (*partner*)	
• CIHR Institute of Indigenous Peoples’ Health (*partner*)	
• CIHR Institute of Musculoskeletal Health & Arthritis (*partner*)	
• CIHR Institute of Neurosciences, Mental Health, & Addictions (*partner*)	
• CIHR HIV/AIDS Research Initiative (*partner*)	
• Alberta Innovates–Health Solutions (*partner*)	
• Fonds de recherche du Québec–Santé (*partner*)	

**Box 2** 2014 cohort of Applied Public Health Chairs

**Table:** 

Thematic foci of research programs	
• Population health and HIV prevention (Paula Braitstein, University of Toronto)	
• E-Health and public health interventions (David Buckeridge, McGill University)	
• Canada’s health care system and public health interventions (Damien Contandriopoulos, University of Victoria)	
• Physical activity and public health (Guy Faulkner, University of British Columbia)	
• Health adaptation and climate change (James Ford, formerly McGill University)	
• Evaluating smoking and healthy weight policies (David Hammond, University of Waterloo)	
• Urban interventions and public health (Yan Kestens, Université de Montréal)	
• Chronic disease prevention and youth (Scott Leatherdale, University of Waterloo)	
• Aboriginal health equity and obesity (Jonathan McGavock, University of Manitoba)	
• Oral health and policy (Lindsay McLaren, University of Calgary)	
• Sexual and reproductive health (Wendy V. Norman, University of British Columbia)	
• Public policy and community environments (Candace Nykiforuk, University of Alberta)	
• Global health and community health interventions (Valéry Ridde, formerly Université de Montréal)	
• Indigenous health knowledge and information as tools to advance equity (Janet Smylie, University of Toronto)	

More details available at: http://www.cihr-irsc.gc.ca/e/48898.html

## Our proposal

**Fig. 1:**
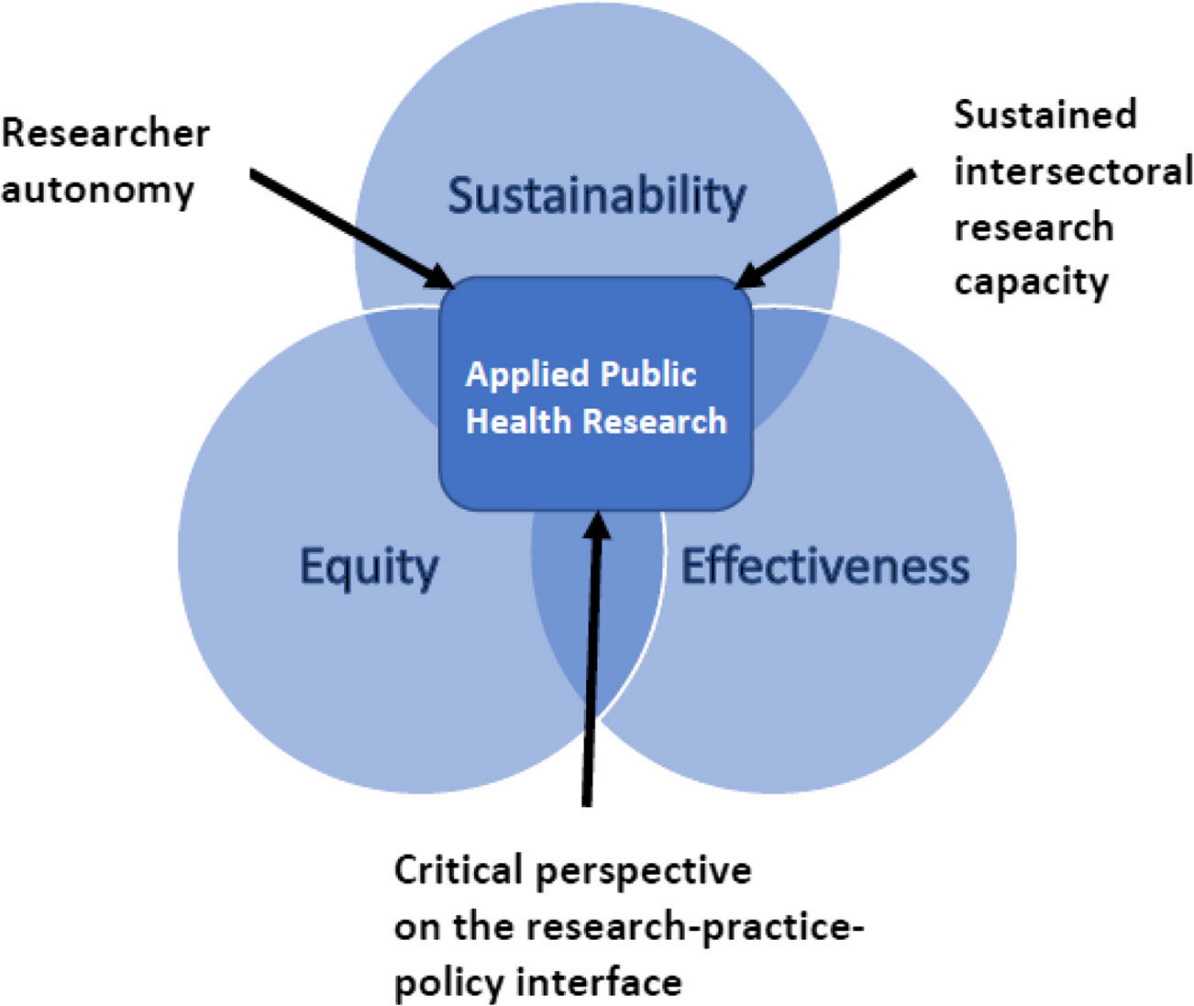
Visual depiction of the role and attributes of applied public health research, vis-à-vis core population and public health principles of equity, sustainability, and effectiveness

We propose that applied public health research is a critical component of a robust population and public health community. As illustrated in Fig. 1, we view applied public health research as sitting at the nexus of three core principles: (1) sustainability, (2) equity, and (3) effectiveness, which align with a vision of public health as critical to a healthy, fair, and sustainable society. By *sustainability*, we mean an approach or way of thinking, about public health in particular (e.g., Schell et al. 2013) and population well-being more broadly (https://sustainabledevelopment.un.org/sdgs) that emphasizes “meet[ing] the needs of the present generation without compromising the ability of future generations to meet their own needs” (Brundtland et al. 1987). Sustainability has social, economic, environmental, and political dimensions. We define *equity* as a worldview concerned with the embedded or systemic—and often invisible—drivers of unfair distributions of health-damaging experiences. In Canada and elsewhere, inequity is entrenched in legacies of colonial, structural racism designed to sustain inequitable patterns of power and wealth. Equity transcends diverse axes and perspectives, and an equity lens is action-oriented (Ridde 2007). Finally,*effectiveness* refers to impact or benefits for population well-being, as demonstrated by rigorous research. Explicit core values (e.g., equity), while important, are insufficient without translation to demonstrable outcomes (Potvin and Jones 2011). These core principles—sustainability, equity, and effectiveness—overlap and are mutually reinforcing; for example, the inequitable concentration of power, wealth, and exploitation of resources precludes sustainability.

Although these principles are applicable to the public health community broadly (i.e., including but not limited to researchers), applied public health researchers are uniquely situated to embrace sustainability, equity, and effectiveness when asking questions and generating policy- and practice-relevant knowledge, as illustrated below. Drawing on our collective experiences, we describe three necessary attributes of applied public health research that support our model in Fig. 1: researcher autonomy, sustained intersectoral research capacity; and a critical perspective on the research-practice-policy interface. We assert that applied public health research is best positioned to contribute meaningfully to the principles of sustainability, effectiveness, and equity if the attributes described below are in place.

### Researcher autonomy

Researcher autonomy is a precondition for innovation and independent thinking, and for building and sustaining the conditions for collective efforts. Our working definition of researcher autonomy is the capacity to devote time and energy to activities that, at the researcher’s discretion, facilitate research that embraces principles of sustainability, effectiveness, and equity. Autonomy, beyond the scope of general academic independence, provides the freedom to build and nurture partnerships, and to navigate among universities, health care systems, governments, communities, and across sectors. Effective and respectful partnerships are critical to rigorous intersectoral work and can provide an important platform to discuss systemic forms of inequity (e.g., Olivier et al. 2016; Morton Ninomiya et al. 2017). Recognizing a potential tension around the role of the researcher in an applied public health context, we deliberately selected the word “autonomy,” which we view as conducive to meaningful collaboration (although that may be experienced differently by different researchers), rather than “independence” which can be seen as contrary to such collaboration. Yet despite their importance, the time and resources to form and sustain those relationships are often not accommodated within funding and academic structures.

Autonomy, when coupled with resources and recognition, permits applied public health researchers to balance foundations of public health with current policy relevance. Although many of us have research programs with particular thematic foci (e.g., physical activity, dental health, HIV), autonomy provides space and credibility to connect those focal issues to enduring and evolving problems in public health (e.g., determinants of population well-being and equity), and to inform the contemporary policy context. Examples include research on health implications of neighbourhood gentrification in urban settings (Steinmetz-Wood et al. 2017); using community water fluoridation as a window into public and political understanding and acceptance of public health interventions that are universal in nature (McLaren and Petit 2018); and using innovative sampling methods to identify how census methods can perpetuate exclusion (Rotondi et al. 2017). That latter work, which estimated that the national census undercounts urban Indigenous populations in Toronto by a factor of approximately 2–4, provides impetus to work towards an inclusive system that respects individual and collective data sovereignty, and that is accountable to the communities from whom data are collected.

These implications of autonomy are consistent with calls for greater reflexivity in public health research (Tremblay and Parent 2014).

**Insight**: To strengthen applied public health research in Canada, researcher autonomy – whereby researchers have the credibility and protected time to set their own agendas in partnerships with the communities they serve – must be privileged.

### Sustained intersectoral research capacity

Applied public health research requires funding for resources and infrastructure that are essential to sustain an intersectoral research program, but for which operating funds are otherwise not readily available. Examples include ongoing cohort studies (e.g., Leatherdale et al. 2014), research software platforms (e.g., Shaban-Nejad et al. 2017), meaningful public sector engagement in developing public health priorities, and knowledge translation activities.

Partnerships, also considered under *researcher autonomy* above, are one form of intersectoral research capacity. In applied public health research, having strong partnerships in place permits timely response to research opportunities that arise quickly in real-world settings. Examples in our cohort include instances where researchers were able to mobilize for rapid response funding competitions in areas of environment and health, communicable disease in the global South, and Indigenous training networks, because collaborative teams and potential for knowledge co-creation and transfer were already in place.

**Insight**: A robust applied public health research community requires sustained funding to support foundations of a credible and internationally-competitive research program (e.g., cohort studies, research software platforms, meaningful public sector engagement) that are difficult to resource via usual operating grant channels.

### A critical perspective on the research-practice-policy interface

One barrier to evidence-based policy in applied public health is an assumption that evidence is the most important factor in making policy decisions, versus a more holistic view of the policymaking process where evidence is one of many factors, as discussed in recent work (Fafard and Hoffman 2018; O’Neill et al. 2019; Ridde and Yaméogo 2018).

Applied public health research is ideally positioned to embrace a critical perspective on the research-practice-policy interface. Several recent trends are promising in that regard. These include the following: substantive efforts to bridge public health and social science scholarship (http://www.cihr-irsc.gc.ca/e/50604.html), growing success by Pillar 4 researchers (including applied public health) in CIHR’s open funding competitions (http://www.cihr-irsc.gc.ca/e/50488.html), and the CIHR Health System Impact Fellowship initiative (http://www.cihr-irsc.gc.ca/e/50612.html), which could facilitate the placement of doctoral and post-doctoral academic researchers within the public health system and related (e.g., public, NGO) organizations.

**Insight**: Applied public health researchers are ideally positioned to embrace and model a sophisticated and interdisciplinary perspective on the research-practice-policy interface. To do so, opportunities for researchers (including trainees) to gain skills and experience to navigate the policy context are needed.

## Conclusion

Against the backdrop of discourse about a weakening of public health infrastructure and fracture within the field (Guyon et al. 2017; Lucyk and McLaren 2017), we believe that there is value in working towards a unity of purpose (Talbot 2018). This commentary was prompted by a shared belief that through our experience with the Applied Public Health Chair Program, we have seen a glimpse of what is needed to achieve a population and public health community that is positioned to tackle societal imperatives, which includes an important role for applied public health research, spanning CIHR Pillars 3 and 4. Anchored in principles of sustainability, equity, and effectiveness, we assert a strong need for applied research infrastructure that privileges and supports: researcher autonomy, sustained funding to support foundations of a credible and internationally competitive research program, and opportunities for researchers (including trainees) to gain skills and experience to navigate the policy context. We welcome and invite further discussion and debate.

## Footnotes

1 Under CIHR-IPPH’s mandate, population health research refers to “research into the complex biological, social, cultural, and environmental interactions that determine the health of individuals, communities, and global populations.”

2 Applied may be defined as follows: “put to practical use,” as opposed to being theoretical (https://www.merriam-webster.com/dictionary/applied).

3 For example: https://uwaterloo.ca/compass-system/ (Leatherdale); http://cart-grac.ubc.ca/ (Norman); http://www.healthsystemsglobal.org/ (Ridde).
