# Correction to: Why public health matters today and tomorrow: the role of applied public health research

**DOI:** 10.17269/s41997-020-00398-z

**Published:** 2020-09-02

**Authors:** Lindsay McLaren, Paula Braitstein, David Buckeridge, Damien Contandriopoulos, Maria I. Creatore, Guy Faulkner, David Hammond, Steven J. Hoffman, Yan Kestens, Scott Leatherdale, Jonathan McGavock, Wendy V. Norman, Candace Nykiforuk, Valéry Ridde, Janet Smylie

**Affiliations:** 1 University of Calgary, Calgary, Canada; 2 University of Toronto, Toronto, Canada; 3 McGill University, Montreal, Canada; 4 University of Victoria, Victoria, Canada; 5 CIHR Institute of Population & Public Health, University of Toronto, Toronto, Canada; 6 University of British Columbia, Vancouver, Canada; 7 University of Waterloo, Waterloo, Canada; 8 CIHR Institute of Population & Public Health, York University, Toronto, Canada; 9 Université de Montréal, Montreal, Canada; 10 Children’s Hospital Research Institute of Manitoba, University of Manitoba, Winnipeg, Canada; 11 University of British Columbia, Vancouver, Canada; 12 University of Alberta, Edmonton, Canada; 13 IRD (French Institute For Research on Sustainable Development), CEPED (IRD-Université Paris Descartes), ERL INSERM SAGESUD, Université Paris Sorbonne Cités, Paris, France; 14 University of Montreal Public Health Research Institute (IRSPUM), Montreal, Canada

**Correction to: Can J Public Health**

10.17269/s41997-019-00196-2

The article “Why public health matters today and tomorrow: the role of applied public health research,” written by Lindsay McLaren et al., was originally published Online First without Open Access. After publication in volume 110, issue 3, pages 317–322 the author decided to opt for Open Choice and to make the article an Open Access publication. Therefore, the copyright of the article has been changed to © The Author(s) 2019 and the article is forthwith distributed under the terms of the Creative Commons Attribution 4.0 International License, which permits use, sharing, adaptation, distribution and reproduction in any medium or format, as long as you give appropriate credit to the original author(s) and the source, provide a link to the Creative Commons licence, and indicate if changes were made. The images or other third party material in this article are included in the article’s Creative Commons licence, unless indicated otherwise in a credit line to the material. If material is not included in the article’s Creative Commons licence and your intended use is not permitted by statutory regulation or exceeds the permitted use, you will need to obtain permission directly from the copyright holder. To view a copy of this licence, visit http://creativecommons.org/licenses/by/4.0.

